# DDX49 as a novel prognostic biomarker regulates colorectal cancer cell proliferation through the TIMM44-PI3K-AKT pathway

**DOI:** 10.3389/fonc.2025.1707092

**Published:** 2025-12-08

**Authors:** Baoyu Huang, Zhipeng Yin, Gang Gao, Longhai Li, Miao Yang

**Affiliations:** 1 Department of Gastrointestinal Surgery, Bozhou Hospital of Anhui Medical University, Bozhou, Anhui, China; 2 Department of Neurology, Bozhou Hospital of Anhui Medical University, Bozhou, Anhui, China; 3 Department of Science and Education, Bozhou Hospital of Anhui Medical University, Bozhou, Anhui, China

**Keywords:** colorectal cancer, DDX49, PI3K-AKT, proliferation, TIMM44

## Abstract

**Objective:**

Colorectal cancer (CRC) ranks as the third most common global cancer. This study aims to explore the expression, function, and mechanism of DEAD-box helicase 49 (DDX49) in CRC.

**Methods:**

Pan-cancer data were obtained from The Cancer Genome Atlas (TCGA) database to compare expression differences of DDX49 across 33 types of cancers. Immunohistochemistry (IHC) was used to detect the protein expression levels of DDX49 in CRC. Kaplan-Meier curves and Cox proportional hazards regression model were employed to demonstrate the prognostic value of DDX49. The effects of key molecules on cancer cell proliferation were assessed using a Cell Counting Kit-8 (CCK-8) assay and colony formation assay. Western blot (WB) was employed to measure key molecules in the PI3K-AKT pathway.

**Results:**

Both TCGA data and IHC showed that elevated DDX49 in CRC tumors was correlated with advanced stages and poor prognosis. DDX49 knockdown inhibited proliferation and colony formation in SW480 and HCT-8 cells, suppressing PI3K-AKT pathway activation and TIMM44 expression—reducing AKT phosphorylation. SC79 treatment partially rescued phosphorylated AKT and proliferation. TIMM44 knockdown mimicked these effects, while its overexpression restored AKT phosphorylation and proliferation in DDX49-knockdown cells.

**Conclusion:**

DDX49, a potential prognostic biomarker, promotes cell proliferation by TIMM44-PI3K-AKT pathway, which may offer a target for clinical anti-tumor therapy.

## 1 Introduction

According to data from the International Agency for Research on Cancer (IARC), colorectal cancer (CRC) is the third most common cancer globally (1). Significant differences exist in its incidence and mortality across regions (2). In developed countries such as those in Europe and the United States, CRC has long been highly prevalent (3). For example, in the United States, the incidence of CRC ranks among the top cancers (4). In emerging economies, such as some Asian and African countries, the incidence of CRC has shown a rapid upward trend in recent years (5). Globally, it is estimated that by 2040, the number of new cases will reach 3.2 million, and the number of deaths will increase to 1.6 million, highlighting the severity of the CRC prevention and control (6, 7). The prognosis of CRC is collectively influenced by multiple factors, including tumor biological characteristics, clinicopathological features, treatment protocols, and individual patient status (8, 9). Studies have demonstrated that early diagnosis can significantly improve patient prognosis (10, 11). CRC prognosis correlates directly with tumor stage (such as TNM stages). In stage I-II CRC, the tumor is confined to the mucosal layer or intestinal wall, with no lymph node metastasis or distant spread, allowing high surgical resection rates and a 5-year survival rate exceeding 90% (12). However, in advanced stages (III/IV), the tumor invades deep tissues, lymph nodes, or distant organs (eg, liver and lung metastases). The 5-year survival rate decreases significantly (approximately 45%-75% for stage III, and only about 10% for stage IV) (13, 14). CRC arises from multifactorial and multistage interactions, encompassing genetic, environmental, lifestyle, and gut microbiota factors, among others (15). Therefore, an in-depth exploration of its pathogenesis is of great importance for the diagnosis and treatment of the disease.

The DDX family (DEAD-box protein family) represents a class of ATP-dependent RNA helicases ubiquitously distributed across eukaryotes and prokaryotes, named for their conserved DEAD (Asp-Glu-Ala-Asp) motif (16). Family members exert pivotal functions in virtually all stages of RNA metabolism, including transcription, splicing, translation, degradation, nucleocytoplasmic transport, and ribosome biogenesis, thereby serving as fundamental molecular machinery for orchestrating gene expression (17). To date, approximately 50 members (eg, DDX1, DDX21, DDX3X, DDX5, DDX39B) have been identified, with sequence homology predominantly localized to the conserved helicase domain (18). In contrast, the non-conserved N-terminal and C-terminal regions dictate functional specificity (19). In recent years, accumulating evidence has established a close association between the DDX family and tumor progression. Specifically, DDX5 and DDX17 exhibit elevated expression in a spectrum of malignancies, including breast cancer, colorectal cancer, and pancreatic cancer. These proteins drive tumor progression by facilitating oncogene transcription (eg, c-Myc, EGFR) or inducing dysregulation of splicing factors (eg, SF3B1) (20–23).

The human DDX49 gene resides in the 19p13.11 region of chromosome 19 and comprises 14 exons. As a member of the DEAD-box helicase family, the DDX49 protein, alternatively referred to as DBP8, plays a crucial role in various cellular processes (24). Previous studies have firmly established the significant overexpression of DDX49 across diverse cancer types, strongly indicating its potential involvement in cellular oncogenic transformation (24, 25). However, despite these findings, the role of DDX49 in colorectal cancer remains largely unexplored, with no comprehensive studies conducted to date. To address this gap, this study is dedicated to a thorough investigation of DDX49’s expression, functional implications, and underlying mechanisms in CRC. The ultimate goal is to generate valuable insights that can inform and guide future strategies for the prevention and treatment of CRC.

## 2 Materials and methods

### 2.1 Sources, access and use of public databases

The TCGA dataset was retrieved following the methodology described in previous studies (26, 27). The acquired RNA-seq raw data, presented in FPKM (Fragments Per Kilobase of transcript per Million fragments mapped) format, contains information regarding gene expression levels. First, the SummarizedExperiment package was utilized to extract the DDX49 gene expression matrix; subsequently, the limma package was employed to perform logarithmic transformation of the data, specifically log_2_(FPKM + 1). The R package DESeq2 was used to conduct differential expression analysis of DDX49 on RNA-seq data, comparing tumor tissues with their adjacent normal tissues in both pan-cancer and CRC cohorts. R software (version 4.2.2) and its associated packages were used, with statistical significance defined as *P* < 0.05. All TCGA data utilized in this study were publicly accessible and anonymized, and they complied with the privacy protection standards outlined in the Health Insurance Portability and Accountability Act (HIPAA). Consequently, no additional ethical approval was required.

### 2.2 Immunohistochemical staining assay

The IHC for DDX49 was conducted following the general procedure (28). Patients admitted between 2020 and 2024 were enrolled in this study. All included patients agreed to participate and signed informed consent. The research was conducted in accordance with the Declaration of Helsinki and approved by the Ethics Committee of Bozhou Hospital Affiliated to Anhui Medical University (Approval No.202023). Participants were informed of their right to withdraw at any time and that their data would be kept confidential. Patient surgical specimens were collected by designated staff and subsequently transferred to pathologists for procedures including fixation and embedding. 4-µm-thick paraffin sections were prepared, and IHC was performed using a rabbit-derived primary antibody against DDX49 (Bioss, Woburn, MA, USA; bs-14330R, 1:200) with reference to a relevant study (29). Two pathological experts comprehensively evaluated the IHC results by combining the proportion of stained cells and staining intensity. The staining results were presented with images captured under 40× and 200× magnifications, and the Mann-Whitney U test was used for statistical analysis of the two subgroups. The Ethics Committee of Bozhou Hospital Affiliated to Anhui Medical University reviewed this study in terms of scientificity, feasibility and patient protection, and approved the study (No. 202023).

### 2.3 Clinical correlation and prognostic risk factor analysis

To investigate the role of DDX49 expression levels in disease progression among patients with CRC, an association analysis was initially performed between DDX49 expression and clinical factors, including patient age and tumor stage. In addition, this study focused on identifying key risk factors influencing disease prognosis. First, Kaplan-Meier curves (survival curves) with Log-rank tests were used to visually illustrate survival discrepancies across different subgroups, stratified by age, gender, and disease stage. A Cox proportional hazards regression model was employed for multivariate analysis. Variables with *P* < 0.05 in the univariate analysis were included, and independent prognostic factors were identified using the stepwise regression method, followed by the generation of forest plots to illustrate these prognostic risk factors. R software was used to develop a nomogram, aiming to demonstrate the value of DDX49 in assessing the prognosis of patients with CRC.

### 2.4 Cell culture, virus packaging, and infection

The CRC cell lines HT-29, LoVo, SW480, HCT-8, NCM460 (normal intestinal epithelial cells), and 293T were obtained from the Cell Bank of the Chinese Academy of Sciences. All cells were cultured in high-glucose DMEM medium supplemented with 10% fetal bovine serum (FBS), 100 U/mL penicillin, and 100 μg/mL streptomycin, and incubated in a 5% CO_2_ humidified incubator at 37°C (28). Lentivirus was prepared by transfecting 293T cells, following the method described previously (30). Two days after transfection, the lentivirus-containing medium was harvested and filtered. The target cells to be infected should reach 30%–50% confluency. 1 mL of virus suspension was added to each well of a 6-well plate along with 1 mL of culture medium, and 8 µg/mL polybrene was added simultaneously to enhance infection efficiency. Following positive cell selection with puromycin, cellular RNA and proteins were extracted, and the expression of the target gene was detected using RT-qPCR and Western blot (WB) analyses. The primer sequences for the target gene are shown in **Table 1**.

**Table 1:** List of primer sequences in this study.

Primer name		Sequence (5′-3′)
sh-DDX49-1	Forward	CCGGCGAGGAGCAGATCAAGAAGAAGGATCCTTCTTCTTGATCTGCTCCTCGTTTTTG
	Reverse	AATTCAAAAACGAGGAGCAGATCAAGAAGAAGGATCCTTCTTCTTGATCTGCTCCTCG
sh-DDX49-2	Forward	CCGGCGAGGACTGGTCCATTATCATGGATCCATGATAATGGACCAGTCCTCGTTTTTG
	Reverse	AATTCAAAAACGAGGACTGGTCCATTATCATGGATCCATGATAATGGACCAGTCCTCG
sh-TIMM44-1	Forward	CCGGCGTGGTGTTTAACCGGTTCTTGGATCCAAGAACCGGTTAAACACCACGTTTTTG
	Reverse	AATTCAAAAACGTGGTGTTTAACCGGTTCTTGGATCCAAGAACCGGTTAAACACCACG
sh-TIMM44-2	Forward	CCGGCCTGTTCTCCAAGACAGAGATGGATCCATCTCTGTCTTGGAGAACAGGTTTTTG
	Reverse	AATTCAAAAACCTGTTCTCCAAGACAGAGATGGATCCATCTCTGTCTTGGAGAACAGG
actin beta	Forward	GGCACCCAGCACAATGAAGA
	Reverse	ACTCCTGCTTGCTGATCCAC
DDX49	Forward	GTCATCGTGGCTCGTGGAA
	Reverse	TTCCCACTGCCTGTCTTAGC

### 2.5 RNA extraction and real-time fluorescent quantitative reverse transcription-polymerase chain reaction

Cells were collected and TRIzol reagent was added to extract RNA. Total RNA was extracted following the kit instructions, with reference to a previous study (31). After determining the RNA concentration using a spectrophotometer, 1 µg of total RNA was used for reverse transcription into cDNA with a commercial reverse transcription kit (Thermo Fisher Scientific, Waltham, MA, USA). Using the SYBR Green method, fluorescent signals were monitored in real-time, with β-actin serving as an internal reference to normalize inter-sample variations. This enabled quantitative analysis of the relative cDNA expression levels. Finally, gene expression levels were calculated using the 2^^(-ΔΔCt)^ method based on the fluorescence threshold (Ct value).

### 2.6 Western blot

CRC cells were harvested and lysed in pre-chilled protein lysis buffer (supplemented with protease inhibitors and phosphatase inhibitors). Protein concentration was determined using the bicinchoninic acid (BCA) assay (32). For sodium dodecyl sulfate-polyacrylamide gel electrophoresis (SDS-PAGE), 20 μg of each sample was loaded into each well sequentially. The electrophoresed gel was aligned with a nitrocellulose membrane (NC membrane) for wet transfer at a constant voltage of 100 V for 2 hours. After transfer, the membrane was blocked in 5% BSA solution on a shaker at room temperature for 1 hour, then incubated overnight at 4°C with primary antibodies: anti-DDX49 (1:1, 000, 20523-1-AP; Proteintech, Wuhan, China), anti-β-tubulin (1:5, 000, 10068-1-AP; Proteintech, Wuhan, China), anti-PI3K-p85 (1:1, 000, 60225-1-Ig; Proteintech, Wuhan, China), anti-phosphorylated PI3K-p85 (p-PI3K-p85, 1:1, 000, AF3242; Affinity Biosciences, Cincinnati, OH, USA), anti-PI3K-p110 (1:1, 000, 20584-1-AP; Proteintech, Wuhan, China), anti-phosphorylated-PI3K-p110 (p-PI3K-p110, 1:1, 000, bs-6417R; Bioss, Beijing, China), anti-AKT (1:1, 000, 10176-2-AP; Proteintech, Wuhan, China), anti-phosphorylated-AKT (p-AKT, 1:1, 000, 66444-1-Ig; Proteintech, Wuhan, China), anti-FARSA (1:1, 000, 18121-1-AP; Proteintech, Wuhan, China), anti-TIMM44 (1:1, 000, 13859-1-AP; Proteintech, Wuhan, China), anti-TIMM13 (1:1, 000, 11973-1-AP; Proteintech, Wuhan, China), anti-MRPL4 (1:1, 000, 27484-1-AP; Proteintech, Wuhan, China), and anti-PIN1 (1:1, 000, 10495-1-AP; Proteintech, Wuhan, China). The membrane was washed 3 times with TBST buffer (5–10 minutes per wash), incubated with horseradish peroxidase (HRP)-conjugated secondary antibody at room temperature for 0.5 hours, then washed again. Finally, the membrane was reacted with ECL substrate, and signals were captured using a chemiluminescence imager to visualize target protein bands.

### 2.7 Cell counting kit-8assay

In a 96-well plate, 100 μL of cell suspension (1, 000 cells per well) was added to each well, with each sample tested in triplicate. The cells were cultured in a constant-temperature incubator. Starting the next day, five time points were selected for measurements. For each measurement, 10 μL of CCK-8 reagent was added to each well and incubated for 1 hour. Following incubation, the absorbance (OD value) of each well was measured at 450 nm using a microplate reader (33).

### 2.8 Colony-formation assay

In 6-well plates, cells were digested to prepare single-cell suspensions. After counting, 1, 000 cells were seeded into each well, and the plates were gently swirled to ensure uniform cell distribution. The cells were cultured for approximately 10 days until macroscopic colonies formed. Subsequently, the medium was aspirated, and the cells were fixed with 4% paraformaldehyde, followed by staining with crystal violet. Images were captured for documentation and subsequent analysis (34).

### 2.9 Statistical analysis

In this study, statistical analysis and graphing were performed using SPSS 22.0 software (IBM, Armonk, NY, USA), R 4.2.2 software (R Foundation for Statistical Computing, Vienna, Austria), and GraphPad Prism 9 (GraphPad Software, La Jolla, CA, USA). Data with a normal distribution were expressed as mean ± standard deviation (SD), and comparisons between two groups were performed using the t-test. Non-normal data were presented as median (interquartile range) [M (Q1, Q3)], and the Wilcoxon rank-sum test was used for intergroup comparisons. Count data were expressed as case number (percentage) [n (%)], and comparisons between groups were conducted using Pearson’s chi-square test. Bivariate correlation analyses were performed using Spearman’s test, and the correlation coefficient (r) was calculated. The 5-year survival rate was calculated using the Kaplan-Meier method, and intergroup differences were assessed by the Log-rank test. Prognostic factors were analyzed via a multivariate Cox proportional hazards regression model, with results presented as hazard ratio (HR) and 95% confidence interval (95% CI). All tests were two-tailed, and a *P*-value < 0.05 was deemed statistically significant.

## 3 Results

### 3.1 DDX49 exhibited high expression in CRC

**Figure 1:**
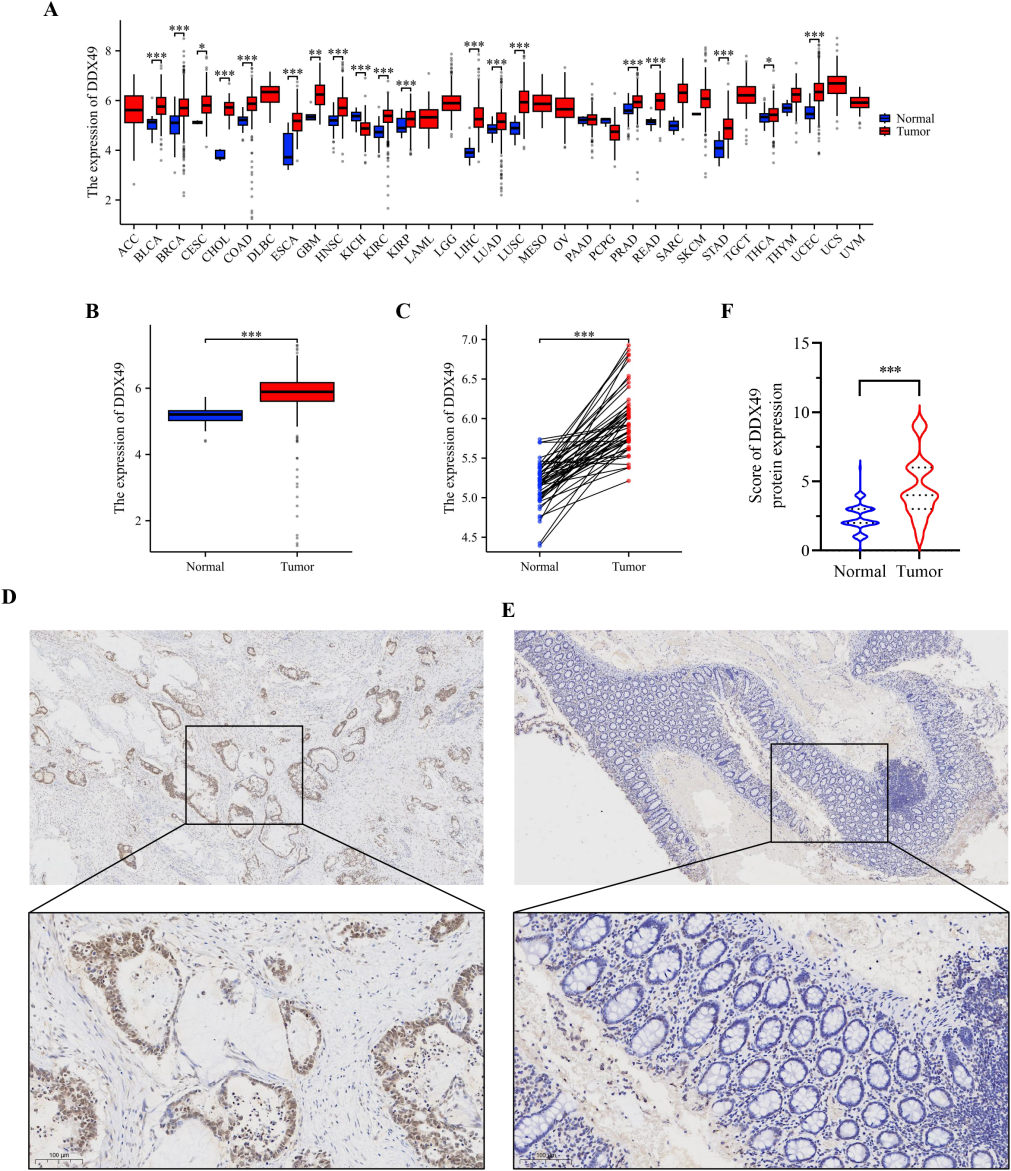
Pan-cancer analysis and IHC results consistently demonstrate that DDX49 was highly expressed in CRC. **(A)** Pan-cancer analysis results of DDX49 (unpaired). **(B, C)** DDX49 expression in CRC (B: unpaired, C: unpaired). **(D)** IHC results of DDX49 in CRC tumor tissues. **(E)** IHC results of DDX49 in CRC paracancerous tissues. **(F)** Comparison of IHC result evaluation scores. *P ≤ 0.05, **P ≤ 0.01, ***P ≤ 0.001, ns, not significant.

Analysis of pan-cancer genomic datasets, including TCGA and GTEx, revealed that the DDX49 gene was aberrantly overexpressed across multiple human malignancies, with transcriptional levels significantly dysregulated in tumor tissues compared to their normal counterparts (*P* < 0.05). Specifically, DDX49 mRNA was upregulated in more than 10 cancer types, such as bladder cancer (BLCA), breast cancer (BRCA), and cervical squamous cell carcinoma (CESC). In other tumor types not explicitly listed, DDX49 demonstrated consistently elevated expression in neoplastic tissues versus adjacent non-tumor tissues (**Figure 1A**). During the comparative analysis of pan-cancer results, DDX49 was identified to exhibit high expression in CRC (**Figure 1A**). Given that CRC has long been a research focus of our group, we further investigated its specific functions and underlying mechanisms in this study.

Next, we analyzed the expression of DDX49 in CRC using the TCGA database. The results showed that DDX49 expression was significantly upregulated in CRC tumor tissues compared with adjacent non-cancerous tissues (**Figure 1B, C**). To visually and accurately assess DDX49 expression in CRC tissues, we subsequently performed IHC staining on clinical specimens from patients with CRC. DDX49 expression in CRC specimens was observed under a light microscope, and staining results were comprehensively evaluated using a scoring system (35). A total of 143 CRC patients were enrolled in this cohort, including 66 females and 76 males. IHC staining results demonstrated that DDX49 expression was significantly higher in CRC tumor tissues than in adjacent normal tissues (*P* < 0.05; **Figures 1D–F**). These findings suggest that DDX49 overexpression may contribute to tumor progression and warrants further investigation.

### 3.2 The expression of DDX49 correlated with clinicopathological features of CRC

**Figure 2:**
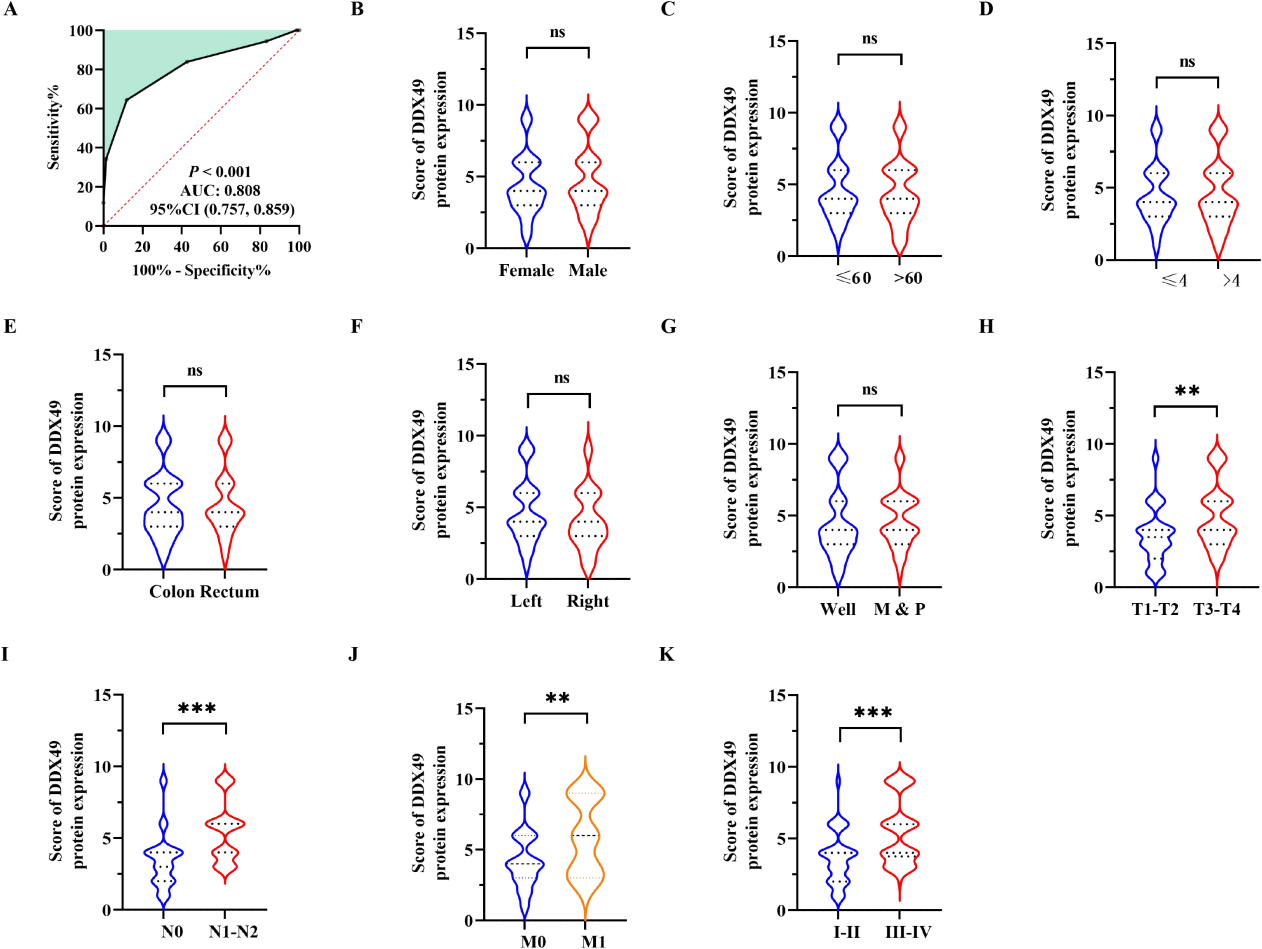
Correlation between DDX49 expression and clinicopathological features in CRC. **(A)** The optimal cutoff value of DDX49 was determined using the Youden index from the ROC curve. **(B)** DDX49 expression in male versus female CRC patients. **(C)** DDX49 expression across different age groups. **(D)** DDX49 expression in relation to tumor size. **(E)** DDX49 expression in relation to tumor location. **(F)** DDX49 expression in relation to cancer site. **(G)** DDX49 expression in relation to histological differentiation (M: moderate differentiation; P: poor differentiation). **(H)** DDX49 expression in relation to depth of tumor invasion (T stage). **(I)** DDX49 expression in relation to lymph node metastasis (N stage). **(J)** DDX49 expression in relation to distant metastasis (M stage). **(K)** DDX49 expression in relation to TNM stage. *P ≤ 0.05, **P ≤ 0.01, ***P ≤ 0.001, ns, not significant.

To clarify the biological function of the potential oncogene DDX49 in the oncogenesis and progression of CRC, this study analyzed the correlation between its expression levels and multiple clinicopathological parameters. Using the receiver operating characteristic (ROC) curve, a cutoff value of 3.5 was determined, and 143 CRC patients were divided into two groups: 92 cases with high DDX49 expression and 51 cases with low DDX49 expression (*P* < 0.05; **Figure 2A**). The results showed that DDX49 expression in CRC tissues was not significantly correlated with parameters such as tumor size, histological grade, and vascular invasion (all *P* > 0.05; **Figure 2B-G**). Notably, DDX49 expression was significantly positively correlated with T stage (T3-T4 vs. T1-T2; *P* < 0.01; **Figure 2H**), N stage (N1-N2 vs. N0; *P* < 0.001; **Figure 2I**), M stage (M1 vs. M0; *P* < 0.01; **Figure 2J**), and TNM stage (III-IV vs. I-II; *P* < 0.001; **Figure 2K**). Further details of the results are presented in **Table 2**. These findings indicate that aberrant high expression of DDX49 may drive the malignant progression of CRC, suggesting its potential role in tumor progression and implying that it may serve as a potential prognostic biomarker for with CRC patients.

**Table 2:** Statistical analysis results of the correlation between DDX49 expression and clinicopathological characteristics in CRC patients.

Characteristics	Case (n=143)	DDX49 expression		χ²	*P*-value
		Low	High		
Total	143	51	92		
Gender				0.149	0.199
Male	76	26	50		
Female	67	25	42		
Age				0.170	0.680
≤60	79	27	52		
>60	64	24	40		
Tumor size				0.153	0.695
≤4cm	62	21	41		
>4cm	81	30	51		
Tumor location				1.180	0.277
Colon	67	27	40		
Rectum	76	24	52		
Cancer site				2.003	0.157
Left	113	37	76		
Right	30	14	16		
Differentiation				3.301	0.069
Well	78	33	45		
Moderate & Poor	65	18	47		
Depth of tumor invasion				6.074	0.014
T1-T2	46	23	23		
T3-T4	97	28	69		
Lymph node metastasis				13.181	< 0.001
N0	69	35	34		
N1-N2	74	16	58		
Distant metastasis				0.540	0.463
M0	122	45	77		
M1	21	6	15		
TNM stage				7.738	0.005
I-II	73	34	39		
III-IV	70	17	53		

### 3.3 Elevated DDX49 was correlated with adverse prognosis in CRC patients

**Figure 3:**
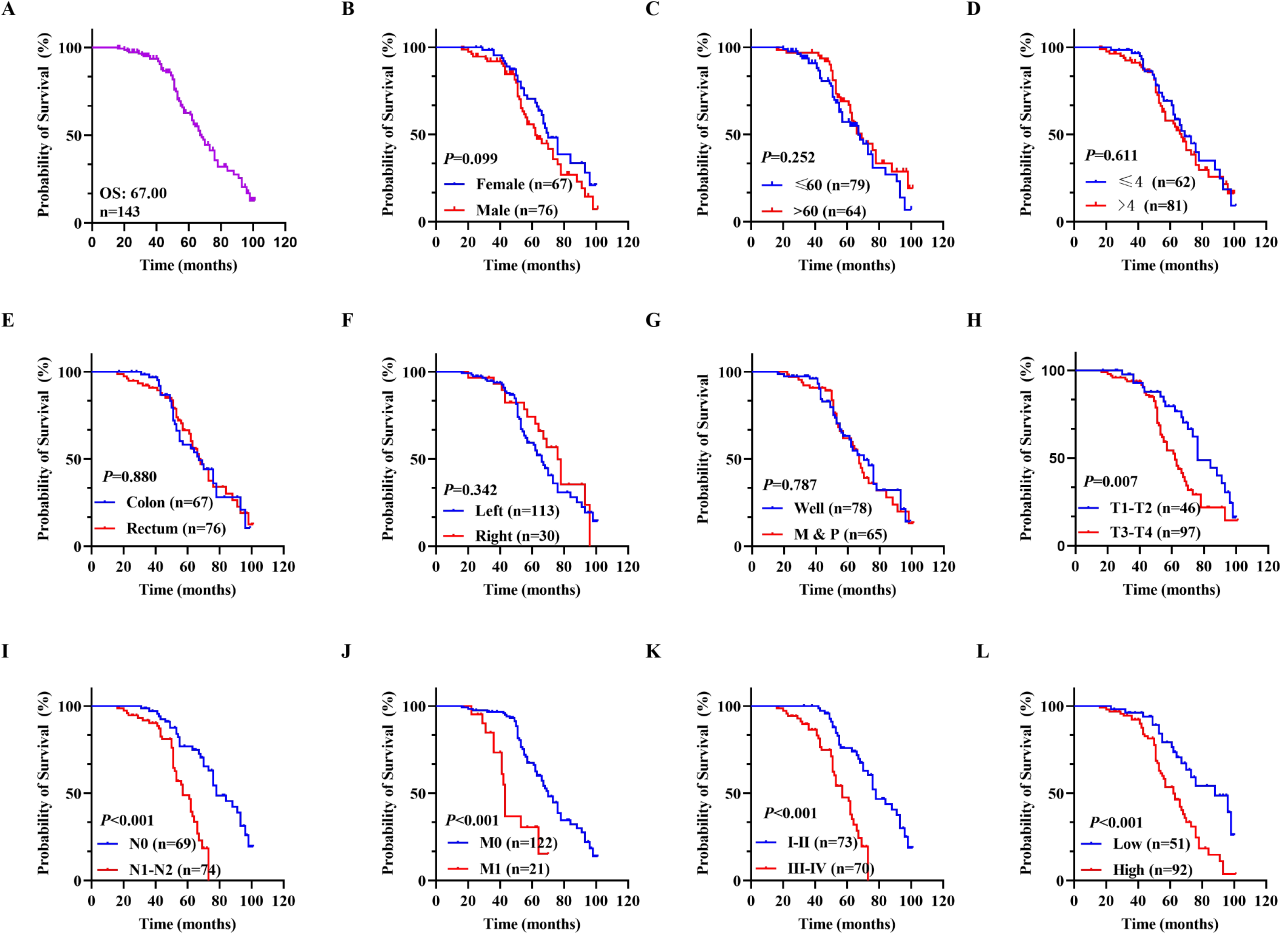
Kaplan-Meier survival curves for OS in CRC patients. **(A)** Overall survival rates in all 143 CRC patients. **(B-G)** Prognostic values of gender, age, tumor size, tumor location, cancer site, and differentiation for OS in patients with CRC, respectively. **(H-K)** Overall survival differences among CRC patients stratified by T stage, N stage, M stage, and TNM stage in sequence. **(L)** Overall survival curves stratified by DDX49 expression. *P ≤ 0.05, **P ≤ 0.01, ***P ≤ 0.001, ns, not significant.

The correlation between gene expression and disease prognosis constitutes a pivotal focus in contemporary medical research. The level of gene expression not only reflects the physiological status of cells but also provides critical insights into disease progression and therapeutic efficacy. In this study, a prognostic analysis of DDX49 expression was performed to investigate its potential value in assessing the survival outcomes of CRC patients. First, the Kaplan-Meier method was employed, and the results indicated that the median overall survival (OS) duration after surgery was 67.00 months, with a 95% CI of 61.51-72.49 (**Figure 3A**). Subsequently, univariate Cox regression analysis showed that T stage (T3-T4 vs. T1-T2) was statistically associated with OS in with CRC patients (HR: 2.003, 95% CI: 1.193-3.363; *P* = 0.009; **Figure 3H**). Similarly, N stage, M stage, and TNM stage were all associated with OS (all *P* < 0.05; **Figures 3I–K**; **Table 3**). Importantly, we found that the high DDX49 expression was associated with poor prognosis (high vs. low, HR: 2.591, 95% CI: 1.333-4.147; *P <* 0.001; **Figure 3L**; **Table 3**). Other variables showed no statistically significant differences (all *P >* 0.05; **Figures 3B–G**; **Table 3**). Furthermore, multivariate Cox regression analysis showed that DDX49 expression (high vs. low) was an independent prognostic factor (HR = 2.351, 95% CI: 1.333-4.147; *P* = 0.003; **Table 3**), indicating that DDX49 expression had a statistically significant independent association with survival outcomes in CRC patients.

**Table 3:** Univariate and multivariate Cox proportional hazards regression analyses in CRC patients.

Clinicopathologic parameters		Median of OS (95% CI)	5-year OS (%)	Univariate analysis		Multivariate analysis	
				HR (95% CI)	*P*-value	HR (95% CI)	*P*-value
Total		67.00 (61.51-72.49)	62.70				
Gender	Female	70.00 (62.58-77.42)	70.30	1.466 (0.922-2.331)	0.106		
	Male	62.00 (61.51-72.49)	56.00				
Age	≤60	67.00 (56.85-77.15)	57.10	0.767 (0.483-1.217)	0.260		
	>60	69.00 (61.14-76.86)	69.20				
Tumor size	≤4cm	69.00 (58.61-79.39)	69.20	1.126 (0.708-1.792)	0.617		
	>4cm	67.00 (60.38-73.64)	56.10				
Tumor location	Colon	67.00 (57.78-76.22)	58.20	0.960 (0.607-1.518)	0.861		
	Rectum	68.00 (59.41-76.59)	64.50				
Cancer site	Left	66.00 (59.59-72.41)	58.00	0.766 (0.438-1.340)	0.351		
	Right	76.00 (66.90-85.10)	74.30				
Differentiation	Well	70.00 (61.07-78.93)	63.20	1.064 (0.673-1.681)	0.790		
	*M-P	67.00 (61.50-72.50)	59.60				
Depth of tumor invasion	T1-T2	76.00 (64.38-87.62)	79.50	2.003 (1.193-3.363)	0.009	1.389 (0.796-2.424)	0.247
	T3-T4	62.00 (55.31-68.69)	52.50				
Lymph node metastasis	N0	78.00 (67.12-88.88)	74.80	3.716 (2.109-6.546)	< 0.001	1.527 (0.455-5.122)	0.493
	N1-N2	57.00 (50.41-63.59)	46.40				
Distant metastasis	M0	70.00 (63.90-76.10)	67.40	3.653 (1.955-6.827)	< 0.001	2.966 (1.463-6.013)	0.003
	M1	43.00 (41.74-44.26)	30.60				
TNM stage	I-II	78.00 (68.37-87.63)	75.90	3.708 (2.147-6.404)	< 0.001	1.615 (0.484-5.385)	0.436
	III-IV	57.00 (51.03-62.97)	47.70				
DDX49	Low	88.00 (66.19-109.81)	76.40	2.591 (1.518-4.422)	< 0.001	2.351 (1.333-4.147)	0.003
High	62.00 (54.73-69.27)	53.40					

*M-P: Moderate-Poor.

**Figure 4:**
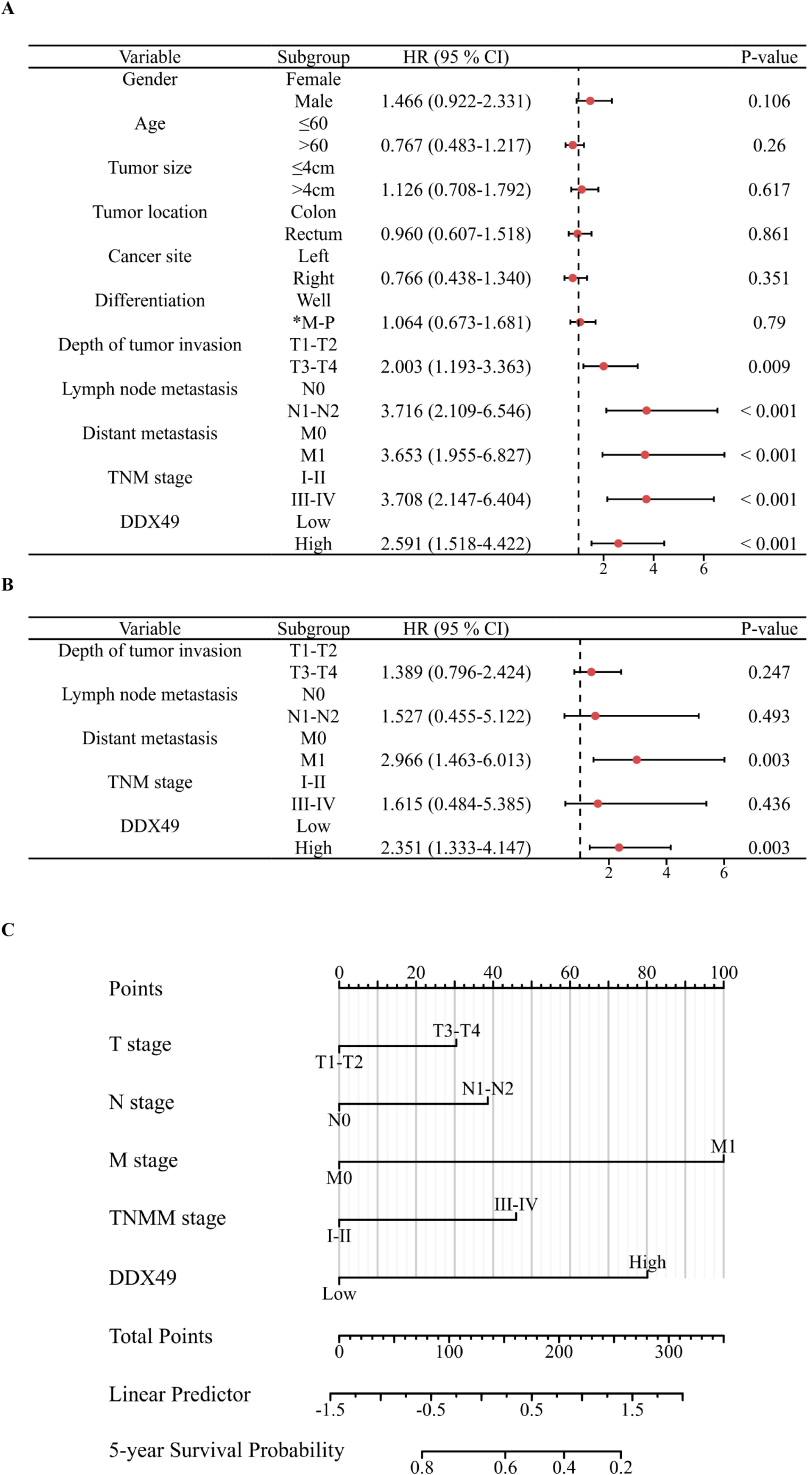
Forest plots and nomogram displaying Cox regression results of prognostic factors associated with overall survival in CRC patients. **(A)** Forest plot of univariate Cox regression analysis. **(B)** Forest plot of multivariate Cox regression analysis. **(C)** Nomogram for predicting 5-year overall survival in CRC patients.

Subsequently, a prognostic forest plot was generated to visualize the magnitude of the impact of risk factors in the Cox regression analysis results. In the forest plots, DDX49 was identified as a prognostic risk factor in both univariate and multivariate analyses (**Figures 4A, B**, **Table 3**). Then, a nomogram was constructed incorporating five variables: T stage, N stage, M stage, TNM stage, and DDX49 expression. Each variable was assigned a specific score based on its respective value, with higher total scores indicating lower 5-year survival probabilities. The results revealed that patients with high DDX49 expression exhibited a poorer prognosis, with a lower predicted 5-year survival rate (**Figure 4C**).

### 3.4 DDX49 exerted facilitative effects on cancer cell proliferation

**Figure 5:**
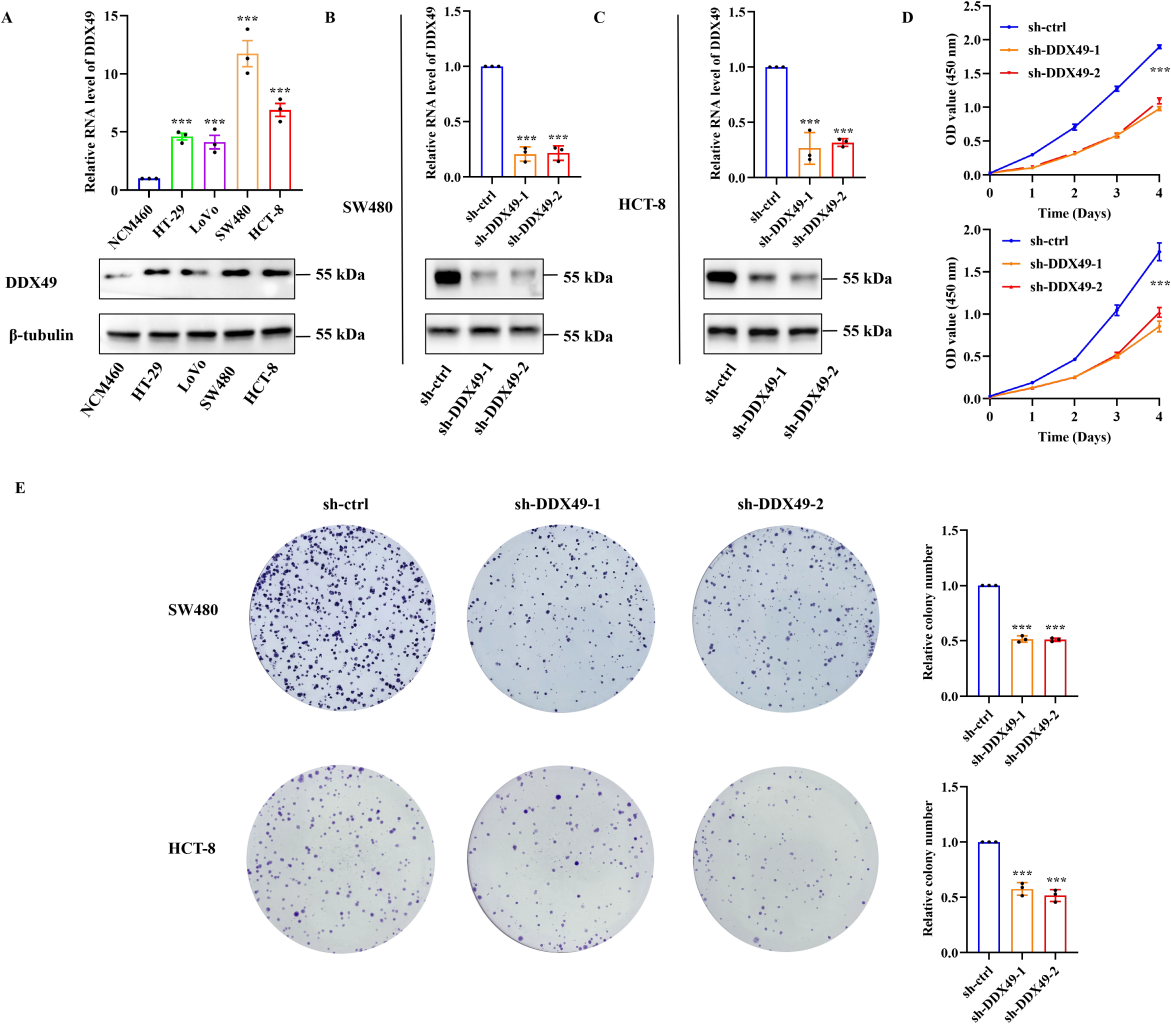
Knockdown of DDX49 significantly inhibited the proliferative capacity of CRC cells *in vitro*. **(A)** Detection of DDX49 expression levels in CRC cells (upper: RT-qPCR, lower: WB). **(B, C)** Validation of knockdown efficiency in DDX49 stably knocked-down cells (left: in SW480, right: in HCT-8). **(D)** Changes in the proliferative activity of CRC cells detected by CCK-8 assay at 0, 1, 2, 3, and 4 days after DDX49 knockdown. **(E)** Representative images of colony formation assay results and bar chart of quantitative analysis of colony-forming efficiency. All experiments were independently repeated at least three times. *P ≤ 0.05, **P ≤ 0.01, ***P ≤ 0.001, and ns, not significant.

Oncogenes are involved in various cellular physiological processes, and their ability to promote cell proliferation is one of the research hotspots. Accordingly, to explore whether DDX49 functions as an oncogene, we investigated the role of DDX49 in tumor cell proliferation using cytological and molecular biology techniques. Given that the expression of the DDX49 gene may be heterogeneous among different CRC cells, we first detected its expression in common cell lines, including HT-29, LoVo, SW480, HCT-8, and NCM460 (normal colonic epithelial cells). The Western blot results were consistent with the mRNA expression patterns: DDX49 was significantly upregulated in CRC cell lines compared with the normal colonic epithelial cell line NCM460 (**Figure 5A**). Notably, its expression levels in HCT-8 and SW480 cells were higher than those in other cell lines, prompting us to select these two cell lines as models for subsequent experiments.

Subsequently, two lentiviral vectors (pLKO.1-puro) harboring DDX49-shRNA were constructed (**Table 1**). Following infection of the target cells, stable DDX49-knockdown cell lines were established via puromycin selection. Validation of knockdown efficiency using qRT-PCR and WB demonstrated high efficiency, thereby enabling subsequent experiments (**Figures 5B****, C**). The CCK-8 assay revealed a significant reduction in the proliferative capacity of sh-DDX49 cells relative to the control group (sh-ctrl group), indicating that DDX49 knockdown effectively inhibited the *in vitro* proliferation rate of CRC cells (**Figure 5D**). Concurrently, colony formation assays demonstrated a marked decrease in the colony-forming efficiency of sh-DDX49 cells, suggesting that DDX49 depletion impaired the long-term proliferative potential of these cells (**Figure 5E**). Collectively, these findings establish that DDX49 may be a critical regulator of cell proliferation.

### 3.5 DDX49 was closely related to the PI3K-AKT signaling pathway in CRC cells

**Figure 6:**
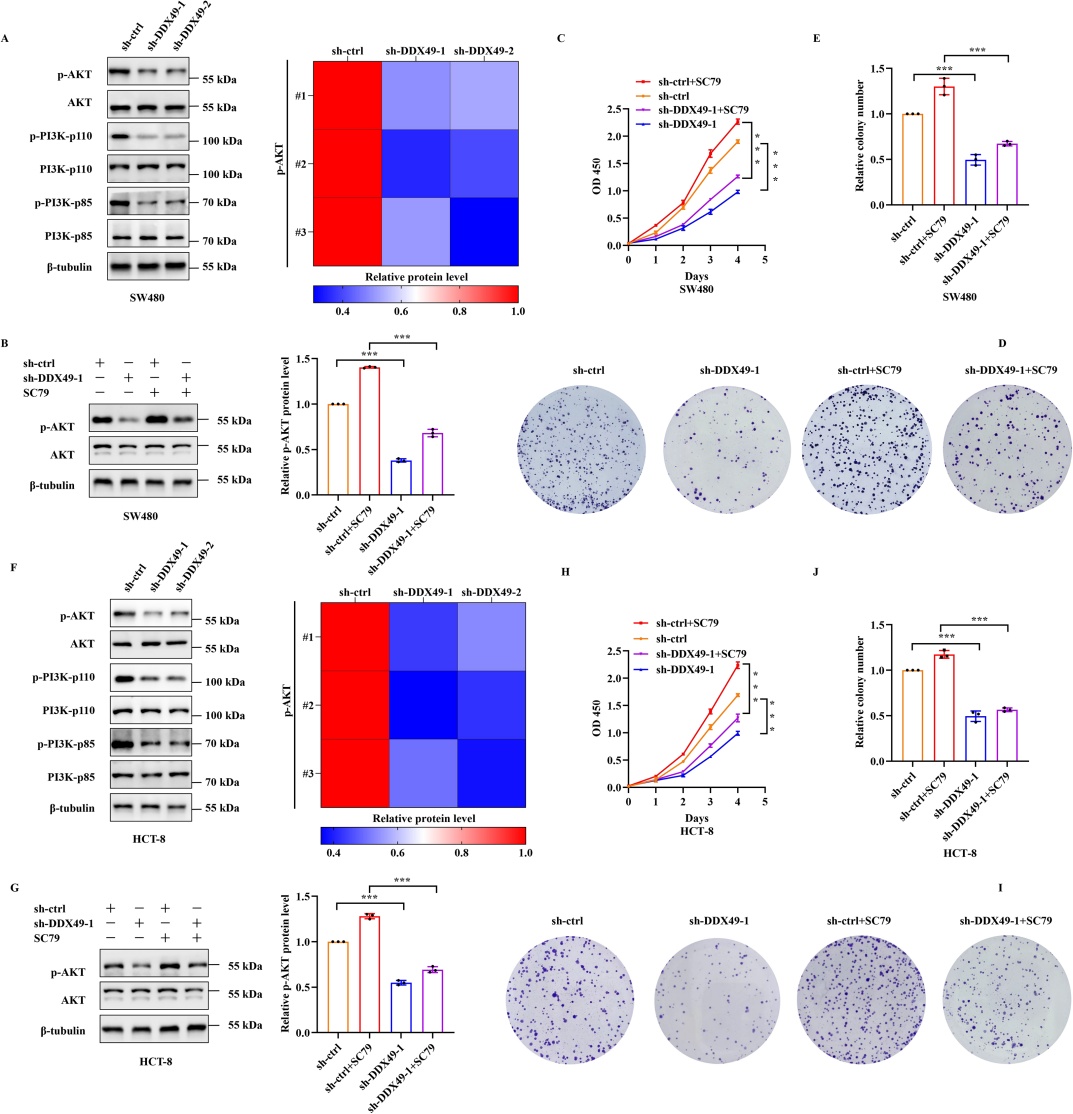
Knockdown of DDX49 led to decreased phosphorylation of the PI3K-AKT pathway in cancer cells. **(A)** Western blot analysis of key PI3K-AKT pathway components in SW480 cells. Left panel (bottom to top, representing molecular weight from high to low): PI3K-p85, p-PI3K-p85, PI3K-p110, p-PI3K-p110, AKT, p-AKT. Right panel: Phosphorylation heatmap. **(B)** Effects of SC79 treatment on AKT phosphorylation in SW480 cells. **(C)** CCK-8 assay measuring cell proliferation in SC79-treated SW480 cells. **(D, E)** Colony formation assays in SW480 cells following SC79 treatment. **(F)** Western blot analysis of PI3K-AKT pathway proteins in HCT-8 cells. **(G)** AKT phosphorylation changes in HCT-8 cells treated with SC79. **(H)** CCK-8-based cell proliferation analysis in SC79-treated HCT-8 cells. (**I, J)** Colony formation ability of HCT-8 cells following SC79 treatment. All experiments were independently repeated at least three times. *P ≤ 0.05, **P ≤ 0.01, ***P ≤ 0.001, and ns, not significant.

In the above results, it has been preliminarily confirmed that DDX49 participates in tumor proliferation; nevertheless, the specific regulatory mechanism underlying this process requires further investigation. Given that numerous members of the DDX family contribute to tumorigenesis via the PI3K-AKT pathway (36, 37), we initially employed western blotting to assess the protein expression levels and phosphorylation status of pivotal components within this pathway—specifically PI3K-p85, PI3K-p110, AKT, and their phosphorylated forms—in DDX49-knockdown cells. WB analysis confirmed that genetic depletion of DDX49 in SW480 and HCT-8 cells did not affect the steady-state protein levels of PI3K-p85, PI3K-p110, or AKT. Nevertheless, phosphorylation levels of all three effectors, especially AKT, were significantly attenuated (**Figures 6A, F**), suggesting impaired PI3K-AKT pathway activation downstream of DDX49. To further clarify the role of the PI3K-AKT signaling pathway in the functional mechanism of DDX49, the cancer cells were subsequently treated with SC79 (38), a specific AKT activator. Exogenous addition of SC79 was able to sustain AKT activation, thereby allowing us to examine whether reactivation of this pathway could rescue the phenotypes observed in DDX49-knockdown cells. The addition of SC79 partially rescued the impairment of phosphorylated AKT and cell proliferation induced by DDX49 knockdown (phosphorylated AKT: **Figures 6B, G**; CCK-8: **Figures 6C, H**; colony formation: **Figure 6D, E, I, J**). The above results indicate that the anti-tumor effect induced by DDX49 knockdown is at least partially mediated by the PI3K-AKT pathway.

### 3.6 Knockdown of the *DDX49* gene leads to significant modulation of TIMM44 in CRC cells

**Figure 7:**
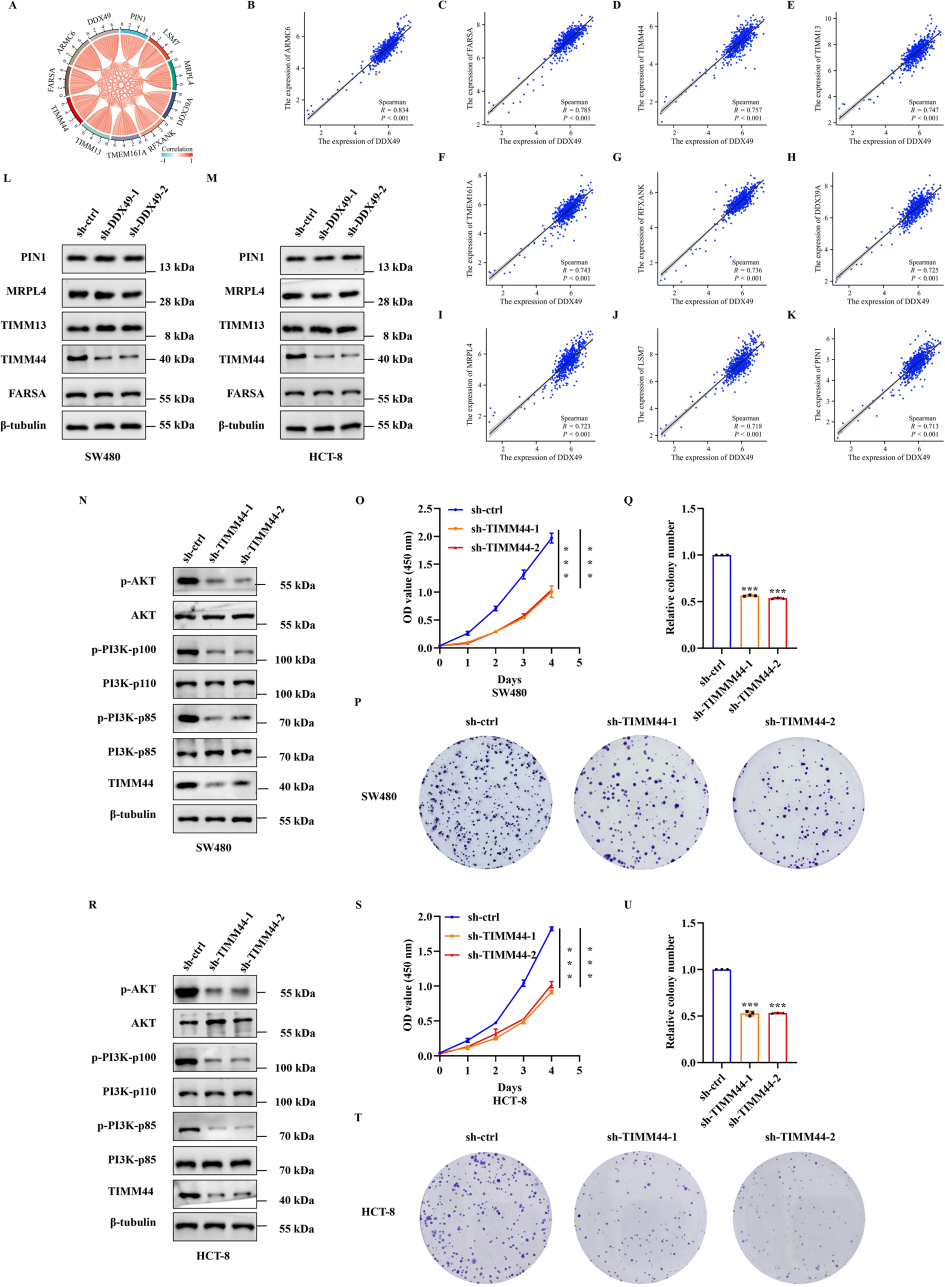
DDX49 could affect the expression of TIMM44 in CRC cells. **(A)** A chord diagram of the top 10 genes correlated with *DDX49*, screened from the TCGA database in CRC. **(B-K)** Scatter plots of correlations between *DDX49* and the ten related genes (From B to K, these genes are as follows: *ARMC6*, *FARSA*, *TIMM44*, *TIMM13*, *TMEM161A*, *RFXANK*, *DDX39A*, *MRPL4*, *LSM7*, and *PIN1*). **(L)** WB analysis validating differential expression of the five DDX49-correlated genes post-knockdown in SW480 cells. **(M)** WB analysis validating differential expression of the five DDX49-correlated genes after knockdown in HCT-8 cells. **(N)** WB assessing PI3K-AKT pathway protein alterations following TIMM44 knockdown in SW480 cells. **(O)** CCK-8 assay results demonstrating the effect of TIMM44 knockdown on SW480 cell proliferation. **(P-Q)** Effect of TIMM44 knockdown on cell colony formation in SW480 cells (P: Stained colony images; Q: Statistical analysis of colony formation efficiency). **(R)** WB assessing PI3K-AKT pathway protein alterations following TIMM44 knockdown in HCT-8 cells. **(S)** CCK-8 assay results demonstrating the effect of TIMM44 knockdown on HCT-8 cell proliferation. **(T-U)** Effect of TIMM44 knockdown on cell colony formation in HCT-8 cells. All experiments were independently repeated at least three times. *P ≤ 0.05, **P ≤ 0.01, ***P ≤ 0.001, and ns, not significant.

To delineate the precise regulatory mechanism by which DDX49 modulates the PI3K-AKT signaling pathway, we performed further investigations. First, leveraging gene expression data from the TCGA public database and tools such as the ‘corrgram’ package in R (39), we generated a chord diagram and scatter plots to visualize the correlations between *DDX49* and its top ten most highly correlated genes. The results indicated that all ten top-ranked genes displayed positive correlations with *DDX49*, with correlation coefficients (r) exceeding 0.7 (all *P* < 0.05, r > 0.7, **Figures 7A–K**). Subsequently, a literature review and relevant research data revealed that five of the ten genes were associated with the PI3K-AKT signaling pathway, including *FARSA*, *TIMM44*, *TIMM13*, *MRPL4*, and *PIN1* (40–44). Therefore, we performed western blotting to assess the protein expression levels of these five genes in DDX49-knockdown cells. Unexpectedly, among the five genes examined, only *TIMM44* showed altered expression levels following DDX49 knockdown (**Figures 7L, M**). Thus, *TIMM44* was subsequently selected as a key focus for further investigations.

While TIMM44 could be regulated by DDX49, its potential to modulate the PI3K-AKT signaling pathway and its impact on cell proliferation in CRC remain unclear, thus meriting further investigation. Building upon these findings, we next knocked down TIMM44 expression in both SW480 and HCT-8 CRC cells to investigate its regulatory role in the PI3K-AKT signaling pathway and the impact on cellular proliferation. Knockdown of TIMM44 resulted in downregulated phosphorylation levels of key molecules within the PI3K-AKT pathway (p-PI3K-p85, p-PI3K-p110, and p-AKT, **Figures 7N, R**), concurrent with diminished cell proliferative activity and a reduction in colony formation numbers (**Figures 7O–Q** and **Figures 7S–U**). Taken together, these findings demonstrated that TIMM44 exerted a significant promotional effect on tumor cell proliferation. At the molecular level, TIMM44 could amplify cell proliferation signals by modulating the activation of the PI3K-AKT signaling pathway, thereby driving abnormal cell proliferation.

### 3.7 DDX49 modulates the PI3K-AKT signaling pathway via TIMM44 to promote cancer cell proliferation

**Figure 8:**
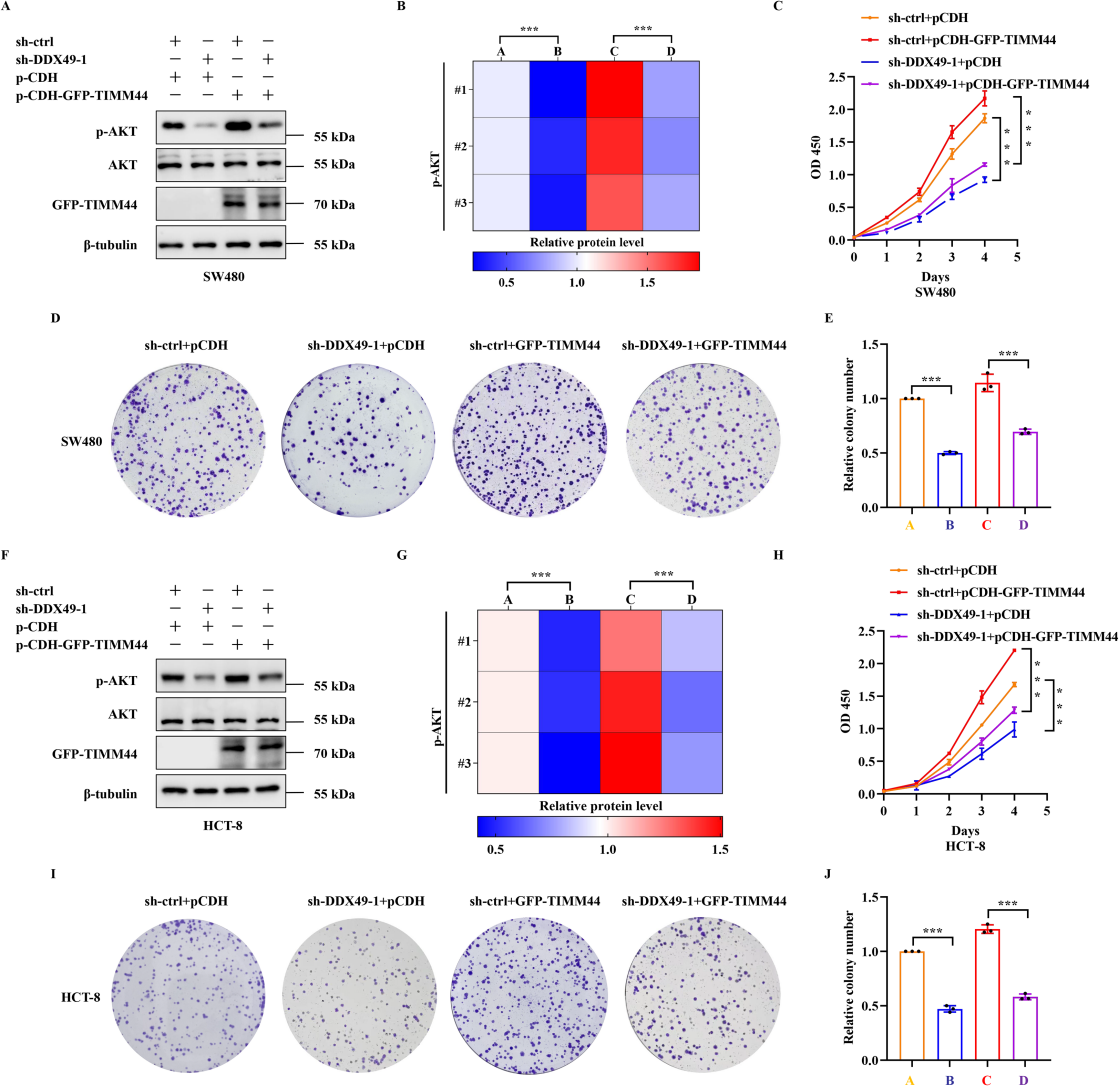
The rescue effect of TIMM44 overexpression on AKT phosphorylation and proliferation phenotype in DDX49-knockdown tumor cells. **(A, B)** WB results and quantitative analysis of phosphorylated AKT (Ser473) and total AKT in different cell groups of SW480. The experimental groups were as follows: sh-ctrl+pCDH (labeled as A in Figure 8B), sh-DDX49-1+pCDH (labeled as B), sh-ctrl+pCDH-GFP-TIMM44 (labeled as C), and sh-DDX49-1+pCDH-GFP-TIMM44 (labeled as D). **(C)** CCK-8 assay showing cell proliferation activity curves of each group over 0–4 days in SW480 cells. **(D, E)** Representative crystal violet staining images and clone count statistics from the colony formation assay in SW480 cells. **(F, G)** WB results and quantitative analysis of phosphorylated AKT (Ser473) and total AKT in HCT-8 cells (labels A-D in Figure 8G correspond to those in Figure 8B). **(H)** CCK-8 assay showing cell proliferation activity curves of each group in HCT-8 cells. **(I, J)** Representative crystal violet staining images and clone count statistics from the colony formation assay in HCT-8 cells. All experiments were independently repeated at least three times. *P ≤ 0.05, **P ≤ 0.01, ***P ≤ 0.001, and ns, not significant.

Preliminary findings have indicated that DDX49 could regulate TIMM44 expression. However, whether DDX49 influences tumor cell proliferation through TIMM44 remains to be elucidated. To address this question, we performed a functional complementation assay, in which TIMM44 was overexpressed in DDX49-knockdown cells, aiming to verify its role in cell proliferation. A key observation from this assay was that overexpression of TIMM44 in DDX49-knockdown tumor cells led to partial recovery of the previously significantly suppressed AKT phosphorylation level. Specifically, the protein expression level of p-AKT (Ser473) was higher compared with that in the DDX49-knockdown group (**Figures 8A, B** for SW480, **Figures 8F, G** for HCT-8). In CCK-8 assays, combined DDX49 knockdown and TIMM44 overexpression restored cell proliferation capacity, which was significantly increased compared to the DDX49 single-knockdown group, although it did not fully reach the level of the control group (**Figure 8C** for SW480, **Figure 8H** for HCT-8). Similarly, concurrent DDX49 knockdown and TIMM44 overexpression resulted in a marked recovery of the cells’ colony-forming ability, with a significant increase compared to the DDX49 single-knockdown group, though it remained lower than that of the normal control group (**Figures 8D, E, I, J**). These findings indicate that TIMM44 overexpression could partially reverse the suppression of cell proliferation caused by DDX49 knockdown, further validating that TIMM44 functions as a downstream mediator in the regulation of tumor cell proliferation by DDX49.

## 4 Discussion

Colorectal cancer ranks among the malignant tumors with persistently high incidence and mortality globally, characterized by a complex pathogenesis involving aberrant regulation of multiple genes and signaling pathways (3, 45). The identification of prognostic biomarkers and key regulatory targets holds significant implications for enhancing the diagnosis and treatment efficacy in patients with CRC. This study focuses on DDX49, an RNA helicase belonging to the DEAD-box family (25), and systematically explores its expression patterns, clinical prognostic value, and regulatory mechanisms underlying tumor cell proliferation in CRC. Through a series of systematic experiments, it could be revealed that DDX49 contributes to CRC progression via the TIMM44-PI3K-AKT pathway, thereby providing novel experimental evidence for prognostic assessment and therapeutic target development in CRC.

Proteins of the DEAD-box family (eg, DDX5 and DDX17) have been demonstrated to play pivotal roles in multiple tumors by engaging in processes such as RNA splicing and translational regulation (20, 46); however, the function of DDX49 in CRC remains elusive. In this study, we observed that DDX49 was highly expressed in CRC tissues, with its expression level significantly correlating with adverse clinicopathological features (including tumor stage and lymph node metastasis) and worse patient survival. Multivariate Cox regression analysis further confirmed that DDX49 could serve as an independent prognostic risk factor for patients with CRC. These findings suggest that DDX49 holds promise as a novel biomarker for assessing the prognosis of patients with CRC. Currently, commonly used clinical prognostic biomarkers for CRC (eg, CEA and CA19-9) have limited specificity. For instance, the AUC of CEA in CRC is mostly 0.66-0.72, whereas the AUC of DDX49 in this study is 0.808 (47, 48). While molecular markers (eg, KRAS and BRAF mutations) incur relatively high detection costs (49, 50). In contrast, as a protein-level marker, differences in DDX49 expression can be detected via routine techniques such as Western blotting and immunohistochemistry, which offer the advantages of simple operation and stable results. Given the relatively small sample size in this study, future validation using large-scale clinical cohorts may enable the combined application of DDX49 with existing markers, thereby enhancing the accuracy of CRC prognostic evaluation (51).

Uncontrolled cell proliferation is a core hallmark of tumorigenesis and progression (52). In this study, *in vitro* functional experiments demonstrated that knockdown of DDX49 significantly suppressed the proliferative activity and colony-forming capacity of CRC cells, suggesting that DDX49 may exert an oncogenic role in CRC. This finding aligns with the proliferation-promoting functions of other DDX family members (eg, DDX10) in CRC, indicating that DEAD-box proteins may regulate the malignant phenotypes of CRC through conserved molecular mechanisms (53). Notably, the proliferation-promoting effect of DDX49 is not restricted to CRC. Recent studies have revealed that DDX49 is also highly expressed in hepatocellular carcinoma and cervical cancer (25, 54), where it promotes cell proliferation, implying that it may act as a pan-cancer oncogene. However, whether its specific mechanisms of action vary across different tumor types remains to be further investigated.

The present study demonstrated that DDX49 could regulate CRC proliferation via the TIMM44-PI3K-AKT pathway. The PI3K-AKT pathway is among the most frequently activated signaling cascades in CRC (55), and its aberrant activation drives tumor growth through mechanisms such as promoting cell cycle progression and inhibiting apoptosis (55–57). However, upstream regulators of this pathway—particularly those mediated by RNA helicases—remain incompletely characterized. Our findings revealed that DDX49 upregulated the expression of TIMM44, a mitochondrial import protein. Elevated TIMM44 expression further activated the phosphorylation of PI3K-p85 and PI3K-p110, and promoted phosphorylation of downstream AKT, ultimately enhancing PI3K-AKT pathway activity (41). The association between DDX49 and the mitochondria-associated protein TIMM44 suggests that DDX49 may indirectly regulate tumor signaling pathways by modulating mitochondrial functions, including energy metabolism and oxidative stress (41, 58).

This study has certain limitations that warrant further investigation. If future conditions permit, our team will pursue the following directions: (1) DDX49 knockout or overexpression models will be constructed in CRC animals models to validate its *in vivo* tumor-promoting effects and regulatory role in the PI3K-AKT pathway. (2) The specific interaction mechanism between DDX49 and TIMM44 will be explored, including aspects such as RNA stability and protein stability. (3) The therapeutic potential of small-molecule inhibitors targeting DDX49 or TIMM44 in CRC will be investigated, thereby providing an experimental foundation for clinical drug development.

In conclusion, this study demonstrates that DDX49 is highly expressed in CRC and correlates with poor patient prognosis, identifying it as a potential prognostic biomarker. Functionally, DDX49 promotes CRC cell proliferation by upregulating TIMM44 to activate the PI3K-AKT pathway. This finding extends our understanding of the role of DDX family proteins in tumors to a certain extent and provides a theoretical foundation for prognostic assessment and targeted therapy in CRC.

## Data Availability

Publicly available datasets were analyzed in this study. This data can be found here: The Cancer Genome Atlas Program (TCGA) - NCI https://www.cancer.gov/ccg/research/genome-sequencing/tcga.
